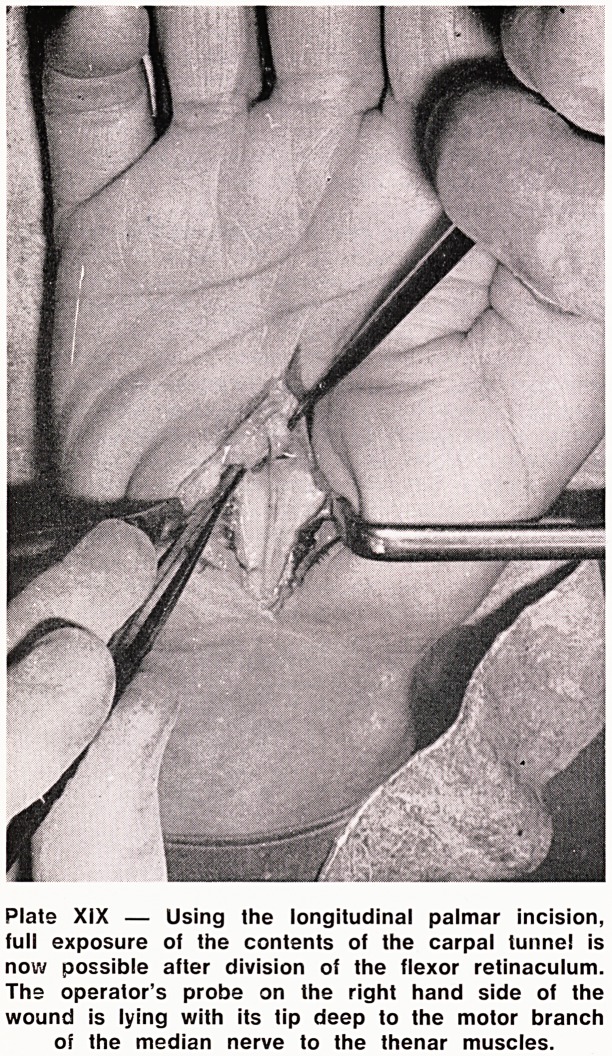# Decompression of the Carpal Tunnel

**Published:** 1971-04

**Authors:** J. E. Woodyard

**Affiliations:** Senior Registrar, Princess Elizabeth Orthopaedic Hospital, Exeter


### Decompression of the Carpal Tunnel

**J. E. Woodyard, F.R.C.S.**

Senior Registrar, Princess Elizabeth Orthopaedic Hospital, Exeter

Carpal tunnel syndrome is the commonest cause of acro-paraesthesia in the upper limb and decompression of the median nerve is therefore a common minor operation. On the whole, the procedure has a good reputation for relief of symptoms and freedom from complications, though not in every series has it been trouble-free (Table 1). This paper reports a review of short-term and a random selection of longer-term follow-ups from this hospital. A brief account of the findings in this series will follow, and from this, certain conclusions can be drawn which may help to improve the surgical management of this condition.

The diagnosis was originally made upon the clinical finding, in particular, the presence of night-time paraesthesia. In some cases, night cock-up splints had been used to confirm the diagnosis by their relief of symptoms.

There were 151 patients, all operated on under a general anaesthetic with a tourniquet. The male to female ratio was 18 to 133, and the majority of patients were between 40 and 60 years of age. 42 patients had bilateral operations making a total of 193 operations in 151 people.

**RESULTS**

When examined personally between 5 months and 6 years after operation they were classified as "satisfactory" if there was complete relief of night pain and tingling, little or no numbness or paraesthesia related to the scar, little or no objective digital numbness and little or no weakness of thumb abduction. The patient was always satisfied but in some cases, the surgeon had reservations. There were 170 satisfactory operations in the series of 193.

The remaining 23 hands were "unsatisfactory". Some had no relief at all from their original symptoms. In others though, there was relief of pain and tingling but there was considerable objective numbness in the median nerve distribution, or a troublesome scar, or marked weakness of thumb abduction, or injury to some structure in the palm.

Delay before operation did not appear to influence results (Table II) though not all previous reports would agree with this finding (Semple and Cargill, 1969). Nor do the unsatisfactory results become more or less frequent with the length of follow-up after 6

**TABLE I**

CARPAL TUNNEL DECOMPRESSION

(Some previous Reports)

| AUTHOR | No. of Operations | Good Results | Fair Results | Poor Results | Complications comments |
|---|---|---|---|---|---|
| Kremer et al 1953 | 40 | 37 (92.5%) | — | 3 (7.5%) | No operative injury. |
| Kendall 1960 | 135 | 123 (98.4%) | — | 2 (1.6%) | No operative injury. |
| Crow 1960 | 42 | 35 (85%) | 5 (12.2%) | 2 (4.8%) | One damaged median nerve. |
| Garland et al 1963 | 109 | 85 (87%) | 22 (19.5%) | 2 (2.5%) | Angled incision recommended. Multiple causes of fair and poor results. |
| Stevenson 1966 | 120 | 55% | 34% | 11% | Six painful scars in elongated or Lazy S incisions. |
| Semple & Cargill 1969 | 150 | 112 (75%) | 38 (25%) | | Transverse scars most frequently tender. Better results with short pre-operative history. 3 cases of injury to digital nerves from inadequate exposure of median nerves. |

### TABLE II

RELATION OF PRE-OPERATIVE HISTORY TO RESULT OF OPERATION

| Length of history | Less than 6 months | 6 - 12 months | More than a year | Total |
|---|---|---|---|---|
| SATISFACTORY | 25 (86%) | 41 (89%) | 104 (89%) | 170 |
| UNSATISFACOTRY | 5 (14%) | 5 (11%) | 13 (11%) | 23 |
| TOTAL | 30 | 46 | 117 | 193 |

months. Recurrence or persistence of symptoms, if it occurs at all, declares itself very early after operation.

Thirteen cases had had delayed healing or haematomata in their wounds and painful and numb scars were seen in no less than 41 hands though by no means all the patients complained much about them. Troublesome scars were twice as common where a transverse wrist crease incision or a longitudinal incision from palm to lower forearm was used. However, even the least troublesome incisions, palmar, longitudinal or angled (Plates XV and XVI) caused some symptoms in 23% of hands.

**Plate XV — The angled skin crease incision recommended for more inexperienced surgeons.**

**Plate XVI — Longitudinal palmar incision which may sometimes be entirely within a skin crease.**

Objective numbness seldom persisted beyond a year unless the whole median nerve distribution was affected. However, in nine of ten such hands, the pre-operative delay had been over six months.

Thumb abduction weakness, or wasting of the thenar eminence was seen in 45 of the 193 hands, and did not clearly relate to the pre-operative delay. Its frequency did not diminish with the length of follow-up, conforming to the usual behaviour of prolonged compression neuropathy of motor fibres.

Results of bilateral decompression were as satisfactory as unilateral operations. However, 21% of the 61 patients with bilateral symptoms had relief of all symptoms in both hands after operation on one hand alone.

**Associated Conditions :**

Three of the 151 patients were known to have rheumatoid arthritis, and these all did well. A further five patients have developed symptoms of "probable rheumatoid arthritis" (A.M.A. criteria) since operation, an incidence of 3.3% compared to the general incidence of the condition which is about 2% of the adult population. This indicates perhaps that carpal tunnel syndrome is only a very infrequent precursor of rheumatoid arthritis.

There were no other significantly frequent associated conditions.

**Discussion :**

An analysis of the cause of the 23 unsatisfactory results shows that these come under five headings :

1. **Incorrect Diagnosis :** certainly the case in two patients (cervical spondylosis and flexor tenosynovitis in the fore-arm) and possibly in two others.
2. **Other painful local conditions :** osteo-arthritis of the wrist and osteo-arthritis of the 1st carpometacarpal joint in two patients.
3. **Severe pain or tingling in the scars :** Three patients complained almost entirely about this, though many others had some lesser paraesthesia.
4. **Injuries to structures in the palm :** Three cases. The median nerve, the tendon of flexor pollicis longus and a digital branch of the median nerve were each divided once.
5. The remainder of the patients with unsatisfactory results were still apparently suffering from the carpal tunnel syndrome to a greater or lesser degree, possibly due to incomplete division of the flexor retinaculum.

The success of the operation appears to depend not only upon correct diagnosis of the cause of symptoms but also upon an adequate and atraumatic exposure and division of the flexor retinaculum, and finally upon healing to give a painless and inconspicuous scar.

It is suggested that the following points of operative technique are worthy of consideration. Whether the operation is performed under general or local anaesthesia, a tourniquet is essential; the angled incision as shown in Plate XV is recommended for the more junior surgeon but with experience, may be curtailed to a longitudinal cut (Plate XVI). The flexor retinaculum should be divided by cutting down under direct vision on to a probe or blunt dissector passed up the carpal tunnel and pressed over to its ulnar side to avoid injuring the motor branch of the median nerve (Plates XVII

**Plate XVII — Using the angled incision, excellent exposure is possible with a little light retraction. The tip of the probe just lies under the proximal edge of the flexor retinaculum.**

**Plate XVIII — With the longitudinal palmar incision, exposure is no more than adequate and requires firm retraction. The tip of the probe lies deep to the flexor retinaculum, the proximal part of which had already been divided.**

**Plate XIX — Using the longitudinal palmar incision, full exposure of the contents of the carpal tunnel is now possible after division of the flexor retinaculum. The operator's probe on the right hand side of the wound is lying with its tip deep to the motor branch of the median nerve to the thenar muscles.**

XIX). Any undivided strands of the retinaculum may be felt by a little finger passed up the tunnel and they also should be divided under direct vision. The tunnel may then be palpated to check there is no local cause for pressure, e.g. ganglia, lipoma of median nerve or inflammation of the synovial sheaths. Only the skin should be closed using carefully-placed sutures to ensure an excellent apposition of skin edges (especially the epidermal layer). These sutures should be left in from ten to fourteen days as healing is sometimes a little slow.

**CONCLUSIONS AND SUMMARY**

193 decompressions of the carpal tunnel in 151 patients are reported with 88% satisfactory results regardless of whether one or both hands were affected and irrespective of the operative delay or length of time they were seen after operation.

21% of 61 patients with bilateral symptoms had spontaneous relief in the non-operated hand after the first carpal tunnel had been decompressed.

The angled incision gives excellent exposure and is preferred and advised for the more inexperienced surgeon.

Relief of symptoms is most unlikely to be followed by late relapse through some digital numbness and paraesthesia may take several months to go.

Carpal tunnel syndrome was only in three to four per cent of cases the precursor of rheumatoid disease.

**REFERENCES**

1. **Crow, R. S.** (1960). Treatment of the carpal tunnel syndrome; British Medical Journal **1**, 1611.
2. **Garland, H. et al** (1963). Carpal tunnel syndrome. British Medical Journal **1**, 581.
3. **Kendall, D.** (1961). Aetiology, diagnosis and treatment of acro-paraesthesiae in the hands. British Medical Journal **II,** 1632.
4. **Kremer, M.** (1953). Acroparaesthesiae in the carpal tunnel syndrome. The Lancet **II,** 590.
5. **Semple, J. C. and Cargill, A. D.** (1969). Carpal Tunnel Syndrome. The Lancet **1,** 918.
6. **Stevenson, T. M.** (1966). Carpal tunnel syndrome : Proceedings of the Royal Society of Medicine **59,** 824.

## Figures and Tables

**Plate XV f1:**
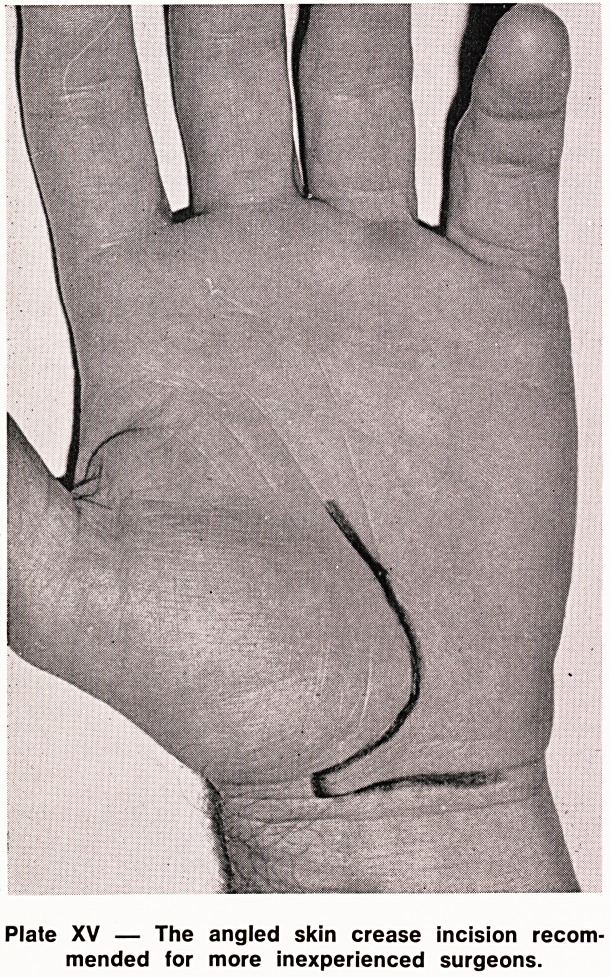


**Plate XVI f2:**
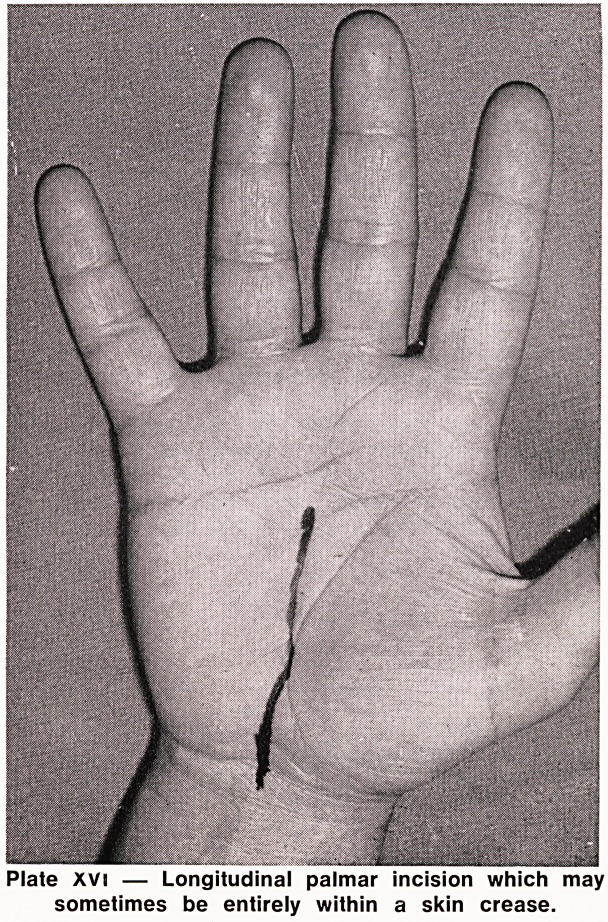


**Plate XVII f3:**
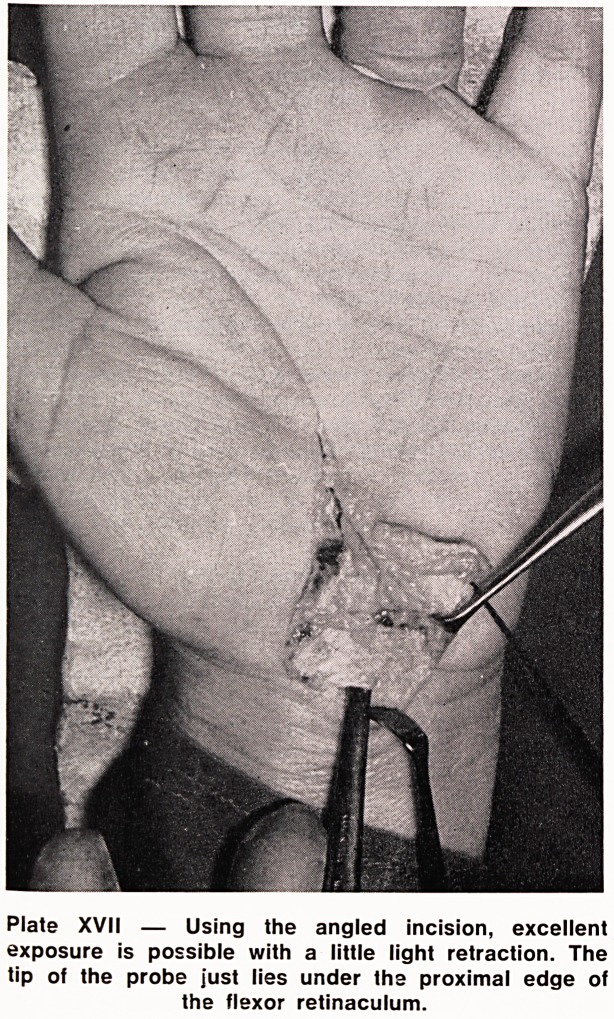


**Plate XVIII f4:**
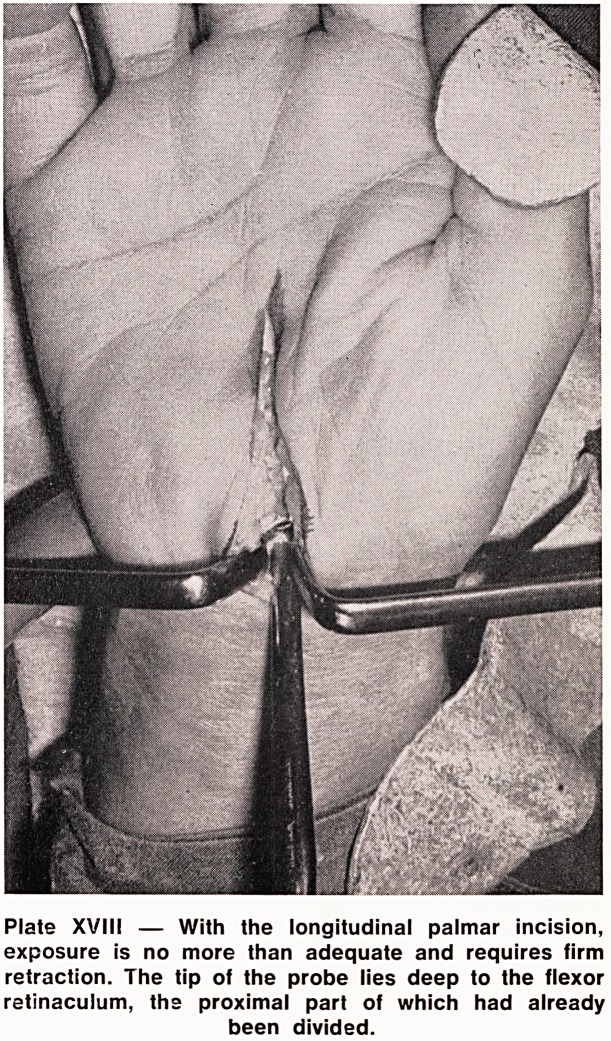


**Plate XIX f5:**